# Clinical Validation of a Highly Sensitive GC-MS Platform for Routine Urine Drug Screening and Real-Time Reporting of up to 212 Drugs

**DOI:** 10.1155/2013/329407

**Published:** 2013-07-10

**Authors:** Hari Nair, Fred Woo, Andrew N. Hoofnagle, Geoffrey S. Baird

**Affiliations:** ^1^Department of Laboratory Medicine, University of Washington, P.O. Box 357110, 1959 NE Pacific Street, Seattle, WA 98185, USA; ^2^Department of Medicine, University of Washington, Seattle, WA 98150, USA; ^3^Department of Pathology, University of Washington, Seattle, WA 98150, USA

## Abstract

An important role of the clinical toxicology laboratory is to provide continuous diagnostic testing for patients with altered mental status and for other medical indications. To meet these needs, we have developed a new Gas Chromatography-Mass Spectrometry (GC-MS) platform that facilitates routine screening and automated reporting of 212 drugs by laboratory technologists around the clock without the need to sign out by an on-site mass spectrometry-trained toxicologist. The platform uses a programmable temperature vaporizer (PTV) injector for large sample volume injection and the free software Automated Mass Spectral Deconvolution and Identification System (AMDIS) for data reduction and spectral matching that facilitates rapid library searching and analyte identification. Method comparison with 118 patient samples demonstrated that this platform and data searching algorithm independently provided improvements in sensitivity compared to an established GC-MS platform. Further examination of the role of the data processing software and the in-house databases used in the established versus the new platform demonstrated that the improved analytical sensitivity of the new platform was attributed to both the technical superiority of the new GC-MS instrumentation and the use of AMDIS in conjunction with the newly generated in-house library for data processing.

## 1. Introduction

Screening patients for toxins, including prescription and/or illegal drugs, is a key function of a clinical toxicology lab. In our hospital, we perform comprehensive toxicological screening of a patient population with a high pretest probability of use, abuse, and overdose of prescription, over-the-counter (OTC), and illegal drugs using a combination of immunoassays and Gas Chromatography-Mass Spectrometry (GC-MS). Clinicians are advised to use this comprehensive urine drug screen to evaluate possible accidental or intentional overdose or poisoning, to assess the type of prescribed and/or illicit drugs used by a patient, or to determine the cause of acute drug toxicity. The assay is also used in specific clinical contexts to investigate whether specific drugs are present in a patient's urine before starting a medical procedure. 

Most clinical toxicology laboratories employ one or more of a variety of analytical and immunological approaches such as Liquid Chromatography-Tandem Mass Spectrometry (LC-MS/MS) [1–4], GC-MS [5, 6], and immunoassay (i.e., EMIT) [7] to screen patient urine samples for a variety of drugs. While immunoassays are fast and fully automated, the technique is prone to moderate-to-severe assay interference from structurally similar members of drug families. Therefore, an immunoassay result is only presumptive and not considered confirmatory. Electron Impact Ionization (EI) GC-MS, on the other hand, is the historical gold standard approach for both qualitative identification and quantitation of a large number of drugs in urine, due to its ability to efficiently separate drugs chromatographically and thereafter produce structurally rich mass spectra that are generally unique to a specific drug [8, 9]. LC-MS/MS in a selective reaction monitoring (or multiple reaction monitoring) mode allows for highly specific qualitative drug identification and highly sensitive drug quantitation, but it is difficult, in practice, to design an effective comprehensive screen based on a large number of transitions because many drugs are not adequately resolved by liquid chromatography. This problem has partly been resolved by the use of high-resolution mass analyzers coupled to LC [4], but this approach requires significant expertise in assay design, an extremely expensive mass spectrometer (~5–10 times more expensive than a GC-MS instrument), and usually a specifically trained staff of operators and clinical interpreters. For these reasons, therefore, we chose to develop a highly sensitive EI GC-MS comprehensive drug screen for use in our clinical laboratory, employing programmable temperature vaporization (PTV) [10] sample injection to increase analyte sensitivity and an automated data analysis algorithm based on free software—Automated Mass Spectral Deconvolution and Identification System (AMDIS) [11–13]—to allow operation by generalist clinical technologists. PTV allows the introduction of a sample into a cold system followed by a temperature ramp that selectively evaporates the injection solvent prior to introduction onto the column, instead of direct injection into a hot oxidative environment of the default injector. This, in turn, allows for the injection of larger volume of sample for enhanced sensitivity. We have characterized the platform using 118 patient samples. Although data analysis strategies similar to ours have been demonstrated in metabolomics [14, 15] and environmental applications [16], clinical validation of such an approach has not been reported previously. Our approach encompasses an effective platform for routine toxicology applications.

## 2. Materials and Methods

### 2.1. Experimental

#### 2.1.1. Chemicals and Reagents

Pharmaceutical drugs and reagents were purchased from Sigma-Aldrich (St. Louis, MO, USA), Grace Davison Discovery Sciences (Columbia, MD, USA), and PhytoLab GmbH & Co. KG (Vestenbergsgreuth, Germany).

#### 2.1.2. Urine Samples

Samples used for the method comparison study were accrued from leftover clinical specimens, stored at −20°C, that had been submitted to the laboratory for comprehensive drug screen analysis on a previous GC-MS platform. Urine for comprehensive drug screens was submitted without preservatives. Specimens were used in accordance with procedures approved by the local Institutional Review Board. 

#### 2.1.3. Sample Preparation

Five mL of urine was pipetted into a 13 × 100 mm disposable glass tube. To this, 50 *μ*L of internal standard (0.35 mg/mL of allobarbital and 0.2 mg/mL cyheptamide in methanol) was added and vortexed. The urine/internal standard mixture was transferred to a ToxiLab (Agilent Technologies, Santa Clara, CA, USA) tube and mixed on rotator for 5 min and centrifuged for 5 min at 2500 rpm. The upper organic layer was transferred into a glass 20 mm × 150 mm culture tube to which 50 *μ*L of 0.025 mol/L HCl had been added, and the mixture was vortexed for 10 minutes. The tube contents were evaporated to dryness at 40°C in a water bath under a stream of air (10 minutes). The dried extract residue was reconstituted with 1 mL methanol and vortexed briefly to redissolve the extract. The sample was finally transferred to a labeled 2 mL snap cap vial, capped, and transferred to the instrument autosampler. 

### 2.2. GC-MS

#### 2.2.1. Instrumentation

The GC-MS platform was a Thermo-Scientific ISQ Mass Spectrometer with Trace GC Ultra Gas Chromatograph, TriPlus RSH automated sample injector, and a computer workstation with X-calibur software (Waltham, MA, USA). The GC column was a Restek capillary column, 30 m × 0.25 mm ID × 0.25 *μ*m, Restek Rtx-5MS Cross bond 5% diphenyl-95% dimethyl polysiloxane (Restek, Bellefonte, PA). GC conditions were as follows: PTV splitless injection mode, 3 *μ*L injection; crosslinked methyl silicone, film thickness 330 nm; injection port temperature ramped from initial 65°C for 1.5 minutes, 280°C for 30 s, and then to 300°C for 35 min; helium carrier gas flow-rate 1 mL/min; and column oven temperature programmed from 150 to 300°C at 14.5°C/min, initial 150°C for 20 min, 250°C for 15 min, and then to 300°C for 5 min. The MS conditions were as follows: electron ionization mode, ionization energy 70 eV, ion source temperature 220°C, capillary direct interface 290°C, and full-scan mode *m/z* 33–550, 1 scan/s with a dwell time of 0.2 s. 

#### 2.2.2. Method

Three *μ*L of urine extract was injected into the PTV liner at 60°C for solvent evaporation, and the residue was transferred to the capillary GC column without split by ramping to 280°C. GC column eluents were directly ionized and analyzed by full-scan EI-MS. For validation experiments, samples run on the new platform were compared with the results from the established GC-MS platform used in the clinical laboratory (see Section 2.2.3). The sample preparation and extraction used prior to analysis on the previous HP system were identical to the current procedure.

Carryover from injection to injection was tested using a urine sample spiked with mixtures of drugs containing 50 *μ*g/mL of each drug (high sample). The injection of this mixture of drugs was preceded and followed by the injection of a negative urine sample. Carryover was quantified using mass spectra generated from the negative urine samples injected before and after the high sample. 

#### 2.2.3. Reference GC-MS: Instrumentation and Method

Hewlett Packard 6890 Gas Chromatograph was combined with an HP 5973 MSD Mass Spectrometer. An HP MS ChemStation (DOS series) was used with HP G1034C software version C03.00 (Agilent Technologies, Santa Clara, CA, USA). The GC column was a Restek capillary column, 30 m × 0.25 mm ID × 0.25 *μ*m, Restek Rtx-5MS Crossbond 5% diphenyl-95% dimethyl polysiloxane (Restek, Bellefonte, PA). GC conditions were as follows: Hewlett-Packard 7683 series automated sample injector, 1 *μ*L injection; 5% diphenyl-95% dimethyl polysiloxane; injection port temperature ramped from initial 65°C to 280°C to 300°C; helium carrier gas flow-rate 1 mL/min; and column temperature programmed from 100 to 310°C at 30°/min, initial time 3 min, final time 8 min. The MS conditions were as follows: electron ionization mode, ionization energy 70 eV, ion source temperature 220°C, capillary direct interface 290°C, and full-scan mode *m/z* 33–550, 1 scan/s with a dwell time of 0.2 s.

### 2.3. In-House Library and Data Analysis

Full-scan data files acquired by the GC-MS system from injecting pure compounds in methanol solution were analyzed by the Automated Mass Spectral Deconvolution and Identification System (AMDIS) [11–13] in simple mode. An AMDIS-readable library of 212 drugs was compiled from using the Lib2NIST converter software version 1.0.0.13 included in the NIST MS-search software version 2.0a. For this, mass spectra for each of the 212 drugs were acquired by injecting 1 mg/mL of the drug in neat methanol. The final settings of the deconvolution and search parameters derived from the results of a series of optimization experiments were as follows: width, 12; adjacent peak subtraction, 2; sensitivity, medium; resolution, medium; and shape requirement, medium. Relative retention time (RRT) was calculated for each drug based on the retention time difference between the drug and the internal standard cyheptamide (RT of the drug/RT of internal standard; RRT of cyheptamide = 1). A relative retention time range of ±0.2 minute window was assigned to each drug in the library to account for sample-to-sample variation in retention times. RRT matching was accomplished using a simple Microsoft Excel macro developed in-house. Only the drugs whose retention times matched the preset RRT range are considered reportable.

### 2.4. Analytical Sensitivity

**Figure 1:**
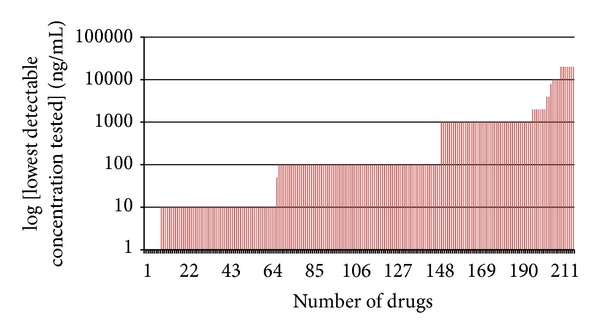
Lowest concentration of each of the 212 drugs detected by the new GC-MS platform. Dilutions of urine containing spikes of drug standards prepared by spiking 1, 10, 100, and 1000 ng/mL into negative urine were used to determine the analytical sensitivity of the platform. If the drug could not be detected at the lowest concentration tested, the next higher concentration of the drug was analyzed. Analytical sensitivity of the drug was recorded as the lowest formulated concentration at which the drug was detected (see Supplementary Material available online at http://dx.doi.org/10.1155/2013/329407 for the list of all drugs along with the lowest concentrations at which each drug was detected).

Analytical sensitivity of the platform was evaluated using dilutions of urine containing spikes of multiple drug standards at various concentrations which were extracted as aforementioned in Section 2.1.3 and injected into the GC-MS. The lowest concentration of a drug in the mixture examined was 1 ng/mL. If the drug could not be detected at the lowest concentration tested, the next higher concentration of the drug was analyzed. In this manner, the concentration of undetected drugs increased in folds (1, 10, 100, and 1000 ng/mL) until the concentration was detectable by the platform.  Figure 1 shows the lowest concentration at which each of the 212 drugs was detected.

### 2.5. Clinical Correlation

Ninety-nine retrospective patient samples were used for clinical correlation of the results generated by the new platform. The list of drugs identified per patient by the new platform was compared with the number of drugs reported by the reference platform for each patient. Discrepancies between the two platforms were adjudicated by chart review, corroborating the analytical results with the patient's history by looking for drugs that were known to have been administered or suspected to have been self-administered based on documented risky behavior. A drug was called positive by the new platform when the analytical criteria (Section 3.1) were met and the clinical records supported the presence of the drug in the patient sample.

## 3. Results and Discussion

### 3.1. Optimization of the New GC-MS Platform

Our objective in introducing a new GC-MS platform in our clinical toxicology laboratory was to enable nonexpert generalist operators to run the assay at all hours, generate reports, and communicate results directly to physicians without the need for verification by a doctorate-level toxicologist with experience in MS-based compound identification. Because the clinical samples for this assay often come from patients with high urine drug concentrations after overdoses, and because the platform was highly sensitive, it was all the more important to evaluate the extent of injection-to-injection carryover first and if necessary to optimize the GC method to minimize carryover (described in 2.2.2). AMDIS parameters were optimized to further ensure that any lingering carryover peaks or potential false positives are excluded from the final report. 

AMDIS extracts pure ion chromatograms from complex compound spectra and deconvolutes noise, peak shape, and retention time and subsequently matches the pure and deconvoluted spectra with those of the in-house reference library (target) consisting of 212 drugs. AMDIS uses two match factors [11, 12], forward search and reverse search, to qualify a match and generate a preliminary list of matched drugs. The AMDIS spectral matches are thus *qualified hits* that can be reported if the RRTs match the preset retention time range in the in-house library. While RRT matching can be performed by AMDIS using a library of external standards (a mixture of commercially available alkanes), such an approach would significantly interfere with the current quality control and daily operating procedures in our laboratory, and it would likely require that RRT bounds be redefined if the GC column needed to be shortened. Thus, we chose instead to assess an acceptable RRT for each individual drug relative to a single internal, rather than external, standard so that laborious external recalibrations were not needed.

Using the optimized GC-MS parameters, data was generated for 99 retrospective patient samples. Using these data, AMDIS deconvolution settings for resolution and sensitivity were first optimized such that maximum numbers of target drugs were generated using a low minimum match factor (MMF) of 60 for both forward and reverse search approaches. At an MMF of 60, some low-level carryover peaks as well as potentially false positive drugs were detected in some samples. Next, the optimal MMF was identified that excluded low-level carryover peaks as well as any false positives that may be present in the report. For this, a range of MMFs (60, 70, 80, and 90) were examined; an example of which is shown in Table 1 for one patient. Table 1 lists the number of drugs reported using each of the 4 MMFs examined. Based on the evaluation of a number of datasets and correlating the results with patient's clinical and prescription histories, it was observed that reports generated with a combined MMF of 80 (or higher) contained no verifiable false positive results. Therefore, an MMF of 80 was used for further validation of the new GC-MS platform.

### 3.2. Clinical Validation: Retrospective Study

**Figure 2:**
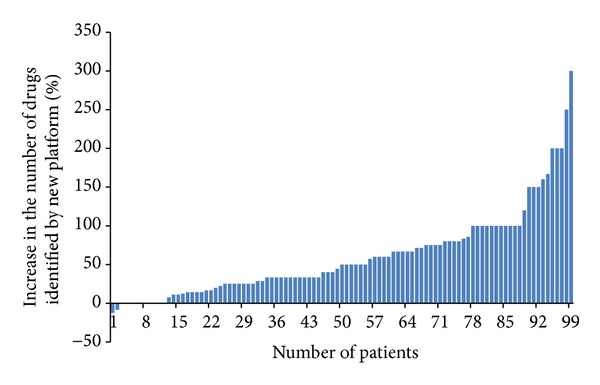
Percentage increase in the number of drugs reported for each patient for the new platform (>0) over the number of drugs reported by the reference platform (<0). The number of drugs reported per patient for the 99 retrospective patient samples was compared with the number of drugs reported for these patients by clinical testing using the reference platform. The % excess (or deficit) in the number of drugs reported by the new platform compared to the reference platform is plotted for each patient. For 2 patients, the reference platform reported one drug each (Clonidine and Clozapine) in excess of the number of drugs reported by the new platform for those patients. For 10 patients, both platforms reported equal number of drugs per patient while for all other patients (*N* = 87) the new platform reported more drugs per patient.

**Figure 3:**
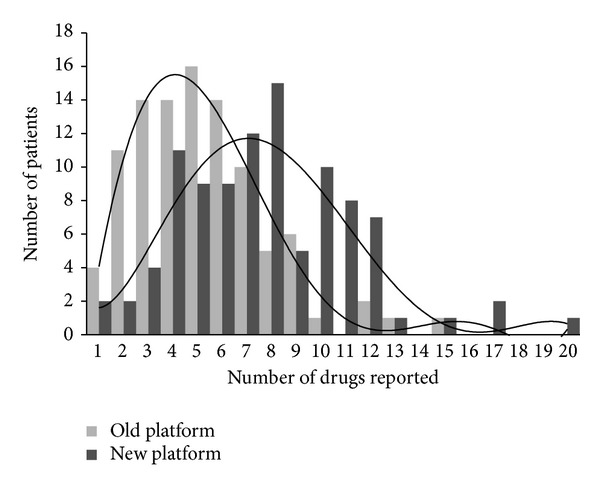
Distribution of qualified hits across 99 retrospective patient samples used for clinical validation. The reference platform reported up to 15 drugs per patient while the new platform reported up to 20 drugs per patient. On an average, the reference platform reported 3–5 patients per sample while the new platform reported 6–8 patients per sample.

Reports were generated for 99 retrospective patient samples using optimized GC-MS and AMDIS parameters. No evidence for false positive test results was observed for any of the 99 reports validated with clinical data. However, in some instances, drugs expected based on the prescription history were not reported at MMF of 80 (see, e.g., Table 1). Because the primary goal of the assay was to ensure that the reported results are true and not to determine every drug present in the patient sample, the extent of false negative results was not examined. The comparison of the number of drugs reported for each of the 99 patients by each platform is plotted in Figure 2. The figure clearly demonstrates that the new platform reported an increased number of drugs per patient compared to the reference platform. In only two patients did the reference platform report more drugs (in fact, a single additional drug) than the new platform. For most patients, the new platform reported more drugs per patient while for a few patient samples both platforms reported equal number of drugs.  Figure 3 shows that for the 99 patient samples the new platform reported, on average, 6–8 drugs per patient while the reference platform reported, on average, 3–5 drugs per patient.

### 3.3. Prospective Study

**Figure 4:**
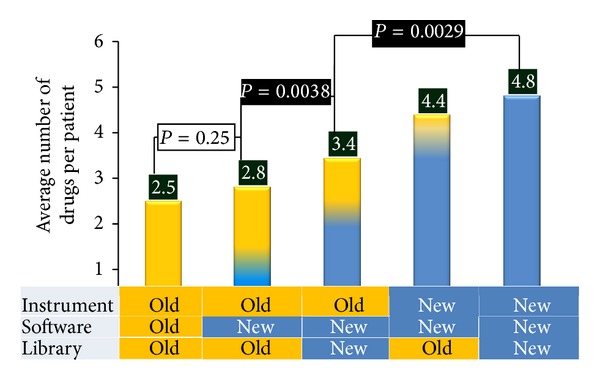
Comparison of the number of drugs identified by the new, reference, and mixed configuration platforms. The number of drugs reported per patient is shown against each configuration, and the statistical significance (*P* value) in the differences in the number of drugs reported for three configurations compared is provided. Statistically valid (*P* value = 0.0038) increment in the number of drugs was reported when the data from the reference GC-MS instrument is analyzed using AMDIS in conjunction with the new in-house library compared to the use of the default data analysis approach. In-addition, both data generated using the reference instrument and that by the new platform were analyzed using AMDIS in conjunction with the new in-house library. The results from this comparison show that an even higher and statistically significant (*P* value = 0.0029) increment in the number of drugs was reported from the data generated by the new platform compared to the data generated by the reference platform.

Because the retrospective study compared data from fresh versus frozen samples and used different urine extracts of the same specimen, a second smaller prospective clinical validation study was performed using 19 fresh patient samples. In this study, drugs were extracted from patient samples for clinical analysis and aliquots of these extracts were analyzed by both the reference platform (for clinical testing) and the new platform (for this study). GC-MS data generated by the new platform and the established platform were processed by their respective software and libraries as well as by compatible configurations of software and libraries across platforms. Five different configurations of analytical components were used in this study and are described in Figure 4 along with the number of drugs reported per patient by each possible configuration of analyzers and software. When data from the reference platform were processed using AMDIS, the number of drugs increased modestly but nonsignificantly (15%,  *P* = 0.25) compared to the number of drugs reported by using the default software for data analysis. However, when the new in-house library, was used in conjunction with AMDIS to analyze data from the reference GC-MS instrument, the number of drugs reported increased significantly (50%,  *P* = 0.0038). When data generated by the new and the reference instruments were both analyzed by AMDIS in conjunction with the new in-house library, a further significant (*P* = 0.0029) increase in the number of drugs (40%) was reported per patient.  Table 2 lists the drugs reported by each of the 5 platform configurations for a subset (three) of patient samples examined by this approach. An increased number of drugs were identified from the data generated by the reference platform when a combination of AMDIS and new in-house library is used. When the data generated by both the new GC-MS instrument and the reference instrument are both processed by AMDIS in conjunction with the new library, the new platform consistently reported a larger number of drugs. The new platform uniquely identified drugs in most patient specimens analyzed (see Table 2). For example, for patient 1 in Table 2, the new platform uniquely identified trazodone, morphine, and gabapentin in the patient's urine, all three of which were missed by the established platform. The patient was prescribed trazodone and gabapentin while he was not prescribed morphine. In this case, the patient who has a history of illicit drug abuse (and hence positive for morphine) was presented to the clinic in altered mental status. Therefore, the physician relied upon the lab results for clinical management. It is conceivable that the knowledge of the presence of these drugs could have altered the management.

Results from both retrospective and prospective studies clearly reveal that the new platform significantly enhanced the range of drugs reported by the toxicology laboratory. Based on the results of the prospective study, we found that the improvement in the clinical performance of the new GC-MS platform resulted partly from the data analysis pipeline but more significantly from the new instrumentation, likely because of the use of PTV injection (allowing increased sample volume and a wider range of drugs to be sampled by GC-MS) and from the improved ion transmission offered by the new quadrupole mass analyzer. Based on our chart review, no verifiable or suspected false positive results were reported for any of the 118 patient samples processed by the new GC-MS platform.

The ability to detect many drugs with our new analytical platform has already aided clinicians in complex toxicology cases. In the case in Table 3, a 71-year-old female patient was admitted to the emergency room for altered mental status. The patient lived in a nursing home and shared a room with another patient who had a similar name. Table 3 shows the list of drugs prescribed to the patient, the list of drugs identified by the new GC-MS platform in her urine specimen, and the list of drugs prescribed to her roommate. As demonstrated in the table, the urine drug screen identified a completely different panel of drugs in her urine compared to the list of drugs prescribed to her. A preanalytical error such as a sample swap was suspected in this case, but the fact that the lengthy drug list derived from the GC-MS analysis matched perfectly to the panel of drugs prescribed to her roommate led the clinical team to determine unequivocally that the cause of the patient's altered mental status was a medication error in the nursing home.

## 4. Conclusion

We have demonstrated that a highly sensitive GC-MS platform employing PTV injection technology for sample injection coupled with a public data analysis package (AMDIS) can be used for routine screening of a large number of drugs in urine in a semiautomated fashion. The newly designed workflow together with a new in-house library of pure compound mass spectra allows for routine analysis of a large number of drugs in urine with superior sensitivity compared to an established platform. By employing higher match quality thresholds, the platform generates qualified (true positive) hits that can be reported in an unsupervised manner by a lab technician around the clock without the need to sign out by an on-site doctoral-level toxicologist. The workflow has been clinically validated for qualitative analysis of urinary drugs and is currently employed for routine toxicological screening of up to 212 drugs in urine. By the use of appropriately designed calibrators, the platform and the analytical approaches described herein can also be used for quantitative analysis.

## Supplementary Material

List of the 212 drugs in the in-house database and the lowest formulated concentrations (ng/mL) at which each drug was detected. These concentrations were determined using dilutions of urine containing spikes of drug standards prepared by spiking 1, 10, 100, and 1000 ng/mL into negative urine.

## Figures and Tables

**Table 1:** List of drugs reported (X) by AMDIS for four MMFs for a patient sample. Number of drugs reported reduced from 15 with an MMF of 60 to 12 (MMF = 70) to 10 (MMF = 80 and 90). Based on such investigation, an MMF of 80 was identified to exclude all verifiable false positives and low-level carryovers from previous injection from the target list.

Target list	60	70	80	90
Methyl salicylate	X	X	X	X
Nicotine	X	X	X	X
Ecgonine methyl ester	X	X	X	X
Cotinine	X	X	X	X
Diphenhydramine	X	X	X	X
Etomidate	X	X	X	X
Levamisole	X	X	X	X
Cocaine	X	X	X	X
Benzoylecgonine	X	X	X	X
Midazolam	X	X	X	X
Propofol	X	X		
Mepivacaine	X	X		
Caffeine	X			
Acetaminophen	X			
Butalbital	X			

**Table 2:** List of drugs identified by the five platform configurations for three patient specimens. More drugs were detected by the new platform compared to the reference platform and the mixed configurations.

	Reference platform	Reference GC-MS with AMDIS and old spectral library	Reference GC-MS with AMDIS and new spectral library	New GC-MS with old spectral library	New platform
Patient 1	Acetaminophen	Acetaminophen	Acetaminophen	Acetaminophen	Acetaminophen
	Caffeine	Caffeine	Caffeine	Caffeine	Caffeine
	Venlafaxine	Venlafaxine	Venlafaxine	Venlafaxine	Venlafaxine
	Codeine	Codeine	Codeine	Codeine	Codeine
	Trimethoprim	Trimethoprim	Trimethoprim	Trimethoprim	Trimethoprim
			Gabapentin	*Morphine *	*Gabapentin *
				*Trazodone *	*Morphine *
				*Theophylline *	*Trazodone *

Patient 2	Nicotine	Nicotine	Nicotine	Nicotine	Nicotine
	Cotinine	Cotinine	Cotinine	Cotinine	Cotinine
	Caffeine	Caffeine	Caffeine	Caffeine	Caffeine
	Diphenhydramine	Diphenhydramine	Diphenhydramine	Diphenhydramine	Diphenhydramine
				*Cocaine *	*Cocaine *

Patient 3	Oxycodone	Oxycodone	Oxycodone	Oxycodone	Oxycodone
	Methadone	Methadone	Methadone	Methadone	Methadone
	Theophylline	Theophylline	Theophylline	Theopylline	Theophylline
	Gabapentin	Gabapentin	Gabapentin		*Gabapentin *
	Caffeine	Caffeine	Caffeine	Caffeine	Caffeine
			Paraxanthine	Paraxanthine	Paraxanthine
				*Morphine *	*Morphine *

**Table 3:** List of drugs detected in the patient specimen, list of drugs prescribed to the patient and the list of drugs prescribed to the patient's roommate. From this comparison it was clear that the patient was administered the drugs of her roommate which potentially caused the altered mental status for the patient.

Drugs detected in the patient specimen by GC-MS	Drugs prescribed to the patient	Drugs prescribed to the patient's roommate
Valproic acid	Fluphenazine	Valproic acid
Desipramine	Benztropine	Desipramine
Citalopram	Atenolol	Citalopram
Clozapine	Lisinopril	Clozapine
Caffeine	Lovastatin	
	Metformin	
	Oxybutynin	
	Cogentin	

## Abbreviations

GC-MS: Gas Chromatography-Mass Spectrometry
PTV: Programmable temperature vaporizer
AMDIS: Automated Mass Spectral Deconvolution and Identification System
OTC: Over the counter
LC-MS/MS: Liquid Chromatography-Mass Spectrometry/Mass Spectrometry
EMIT: Enzyme multiplied immunoassay Technique
RRT: Relative retention time
MMF: Minimum match factor.
