# Supplementary material for: Clinical Validation of a Highly Sensitive GC-MS Platform for Routine Urine Drug Screening and Real-Time Reporting of up to 212 Drugs
Source: J Toxicol. 2013 Jul 10;2013:329407. doi: 10.1155/2013/329407 (PMC3723246; doi:10.1155/2013/329407)
Supplement: Supplementary file 1 — List of the 212 drugs in the in-house database and the lowest formulated concentrations (ng/mL) at which each drug was detected. These concentrations were determined using dilutions of urine containing spikes of drug standards prepared by spiking 1, 10, 100, and 1000 ng/mL into negative urine. [file 329407.f1.pdf]

	Drug/metabolite	Detectable Urine Concentration (ng/mL)
1	10, 11-Carbamazepine Epoxide (Carbamazepine Metabolite)	10
2	1-Benzylpiperazine	2000
3	3-Trifluoromethylphenyl-Piperazine	100
4	6-Monoacetylmorphine (Heroin Metabolite)	50
5	7-Aminoclonazepam (Clonazepam Metabolite)	1000
6	7-Aminoflunitrazepam (Flunitrazepam Metabolite)	100
7	Acetaminophen (Tylenol)	1000
8	Alfentanil	100
9	Alpha-Hydroxyalprazolam (Xanax Metabolite)	10000
10	Alprazolam (Xanax)	10
11	Amantadine (Symmetrel)	1000
12	Amitriptyline (Elavil)	100
13	Amobarbital (Amytal)	10
14	Amoxapine (Ascendin)	100
15	Amphetamine	100
16	Amphetamine Artifact	10
17	Aprobarbital	100
18	Atropine	100
19	Barbital	100
20	Benzoylecgonine (Cocaine Metabolite)	20000
21	Benztropine (Cogentin)	100
22	Brompheniramine (Dimetapp)	10
23	Buprenorphine	1000
24	Bupropion (Wellbutrin)	100
25	Bupropion Metabolite	100
26	Buspirone	1000
27	Butabarbital	1
28	Butalbital	1
29	Caffeine	1
30	Carbamazepine (Tegretol)	10
31	Carisoprodol (Soma)	10
32	Celecoxib (Celebrex)	10
33	Chlorpheniramine	10
34	Chlorpromazine (Thorazine)	10
35	Chlorpropamide	2000
36	Chlorprothixene	10
37	Citalopram / Escitalopram	10
38	Clomipramine (Anafranil)	10
39	Clonazepam	100
40	Clonidine	1000

41	Clozapine (Clozaril, Leponex)	1000
42	Clozapine Metabolite	1000
43	Cocaethylene	10
44	Cocaine	100
45	Codeine	10
46	Cotinine (Nicotine Metabolite)	100
47	Cyclobenzaprine	10
48	Cyproheptadine	10
49	Delta-9 Thc Cooh	10
50	Desalkylflurazepam (Flurazepam Metabolite)	10
51	Desipramine (Norpramin)	100
52	Desmethyldoxepin (Nordoxepin)	1000
53	Dextromethorphan	10
54	Dextrorphan (Dextromethorphan Metabolite; See Levorphanol)	100
55	Diacetylmorphine (Heroin)	10
56	Diazepam (Valium)	10
57	Diethylpropion	10
58	Dihydrocodeine	10
59	Diltiazem (Cardizem)	10
60	Diphenhydramine / Dimenhydrinate (Benadryl/Dramamine)	10
61	Disopyramide (Norpace, Rythmodan)	1000
62	Doxepin (Sinequan)	10
63	Doxepin (Trans)	10
64	Doxylamine	100
65	Duloxetine	20000
66	Ecgonine Methyl Ester (Cocaine Metabolite)	10
67	Eddp (Methadone Metabolite)	100
68	Ephedrine / Pseudoephedrine	20000
69	Ethinamate	100
70	Ethosuximide (Zarontin)	10
71	Ethotoin	10
72	Etomidate	10
73	Fentanyl	1000
74	Fluconazole	100
75	Flunitrazepam (Rohypnol)	100
76	Fluoxetine (Prozac)	1000
77	Fluphenazine	10000
78	Flurazepam (Dalmane)	100
79	Gabapentin (Neurontin)	10000
80	Gemfibrozil	100
81	Glutethimide (Doriden)	10
82	Guaifenesin/Methocarbamol	100
83	Haloperidol (Haldol)	1000
84	Hydrocodone	100

85	Hydromorphone (Dilaudid)	1000
86	Hydroxyzine (Atarax, Vistaril)	1000
87	Ibuprofen (Advil, Motrin)	100
88	Imipramine (Tofranil)	10
89	Jwh-018 [1-Pentyl-3-(1-Naphthoyl)Indole]	1000
90	Ketamine	10
91	Lamotrigine	1000
92	Levamisole	10
93	Levetiracetam (Keppra)	100
94	Levorphanol/Dextrorphan (Optical Isomers)	100
95	Lidocaine (Xylocaine)	10
96	Lorazepam (Ativan)	1000
97	Loxapine	100
98	Maprotiline (Ludiomil)	100
99	Meperidine (Demerol)	1
100	Mephobarbital	100
101	Mepivacaine	100
102	Meprobamate (Equanil, Miltown)	100
103	Mescaline	2000
104	Metaxalone	10
105	Methadone (Dolophine)	100
106	Methadone Metabolite (Eddp)	100
107	Methamphetamine	100
108	Methaqualone (Quaalude)	10
109	Methcathinone	1000
110	Methocarbamol (Robaxin)	2000
111	Methsuximide	10
112	Methyl Salicylate	1
113	Methylenedioxyamphetamine (Mda)	1000
114	Methylenedioxymethamphetamine (Mdma)	100
115	Methylenedioxyprovalerone	10
116	Methylphenidate (Ritalin, Concerta)	10
117	Methyprylon	10
118	Metoclopramide (Reglan)	100
119	Metoprolol (Lopressor)	2000
120	Metronidazole (Flagyl)	100
121	Midazolam (Versed)	10
122	Mirtazapine	10
123	Morphine	1000
124	N-Acetyl Procainamide (Napa)	1000
125	Nalbuphine	1000
126	Nalorphine	100
127	Naproxen (Aleve, Naprosyn)	1000
128	Naproxen Metabolite	20000

129	Nicotine	100
130	Norbuprenorphine	2000
131	Nordiazepam	1
132	Norfentanyl	100
133	Norfluoxetine	10000
134	Norketamine	100
135	Normeperidine	1000
136	Normethsuximide	100
137	Norpropoxyphene	100
138	Norpropoxyphene (Isomer 2)	100
139	Nortriptyline (Aventyl)	1000
140	Norverapamil	1000
141	Olanzapine (Zyprexa)	100
142	Oxazepam (Serax)	20000
143	Oxcarbazepine (Breakdown Product)	100
144	Oxcarbazepine (Trileptal, Trexapin)	1000
145	Oxycodone (Oxycontin)	100
146	Oxymorphone (Numorphan)	1000
147	Papaverine	100
148	Para Aminobenzoic Acid	20000
149	Paraxanthine (Caffeine Metabolite)	4000
150	Paroxetine (Paxil)	1000
151	Pentazocine (Talwin)	100
152	Pentobarbital	10
153	Pentoxifylline (Trental)	10
154	Phenacetin	100
155	Phenazopyridine	1000
156	Phencyclidine (Pcp)	1
157	Phenethylamine	1
158	Pheniramine	10
159	Phenmetrazine	1000
160	Phenobarbital	100
161	Phenothiazine	10
162	Phentermine	1000
163	Phenylbutazone	100
164	Phenylpropanolamine	20000
165	Phenytoin (Dilantin)	100
166	Prazepam	10
167	Primidone (Mysoline)	100
168	Procainamide	1000
169	Procaine	1000
170	Prochlorperazine (Compazine)	100
171	Promethazine (Phenergan)	100
172	Propofol	100

173	Propoxyphene (Darvon)	100
174	Propranol (Breakdown Product)	100
175	Propranolol (Inderal)	1000
176	Protriptyline	1000
177	Psilocin	4000
178	Psilocybin	8000
179	Pyrilamine	100
180	Quetiapine (Seroquel)	1000
181	Quinidine / Quinine (Optical Isomers)	1000
182	Ranitidine (Zantac)	1000
183	Remifentanil	100
184	Salicylamide	1000
185	Scopolamine	100
186	Secobarbital	10
187	Sertraline (Zoloft)	100
188	Strychnine	100
189	Sufentanil	100
190	Talbutal	100
191	Temazepam (Restoril)	100
192	Tetrahydrocannabinol	10
193	Theophylline	2000
194	Thiopental (Pentothal)	100
195	Thioridazine (Mellaril)	100
196	Ticlodipne	10
197	Topiramate (Topamax)	100
198	Tramadol (Ultram)	10
199	Trazodone (Desyrel)	1000
200	Triazolam (Halcion)	100
201	Trifluoperazine (Stelazine)	100
202	Triflupromazine	100
203	Trihexyphenidyl (Artane)	100
204	Trimethoprim (Monotrim)	1000
205	Trimipramine	10
206	Tripelennamine	100
207	Valproic Acid (Depakene, Depakote)	1000
208	Venlafaxine (Effexor)	10
209	Verapamil	1000
210	Zaleplon (Sonata)	100
211	Zolpidem (Ambien)	10
212	Zopiclone (Lunesta)	100
